# The speed of sound in skull-mimicking digital phantoms depends on the microstructure

**DOI:** 10.1088/1361-6560/ae1aca

**Published:** 2025-11-13

**Authors:** Samuel Clinard, Taylor Webb, Henrik Odéen, Dennis L Parker, Douglas A Christensen

**Affiliations:** 1 Department of Biomedical Engineering, University of Utah, Salt Lake City, UT, United States of America; 2 Department of Radiology and Imaging Sciences, University of Utah, Salt Lake City, UT, United States of America; 3 Department of Electrical and Computer Engineering, University of Utah, Salt Lake City, UT, United States of America

**Keywords:** transcranial focused ultrasound, ultrasound simulation, k-Wave, acoustic velocity, skull microstructure, digital bone phantom

## Abstract

*Objective.* Transcranial focused ultrasound therapies depend on accurately focusing the ultrasound beam through the skull. Simulated phase aberration correction with properties derived from computed tomography (CT) can partially restore the focus. However, the typical clinical CT resolution (0.5 mm isotropic) cannot resolve the bone microstructure, introducing uncertainty in the velocity relationship to CT Hounsfield units (HUs), which reduces focusing precision. *Approach.* To demonstrate this, we simulated through-transmission measurements through skull-mimicking digital phantoms consisting of cortical bone and marrow with porosities ranging from 0% to 80%. The phantoms comprised spherical marrow pores (0.1–0.6 mm diameter) randomly placed into a cortical background, forming fine-to-coarse microstructures. Using k-Wave, we simulated pulsed and continuous planar sources at four center frequencies (250 kHz, 500 kHz, 750 kHz, 1 MHz). Group and phase velocities are reported for each pore diameter and porosity. The steady-state phase is reported through representative phantoms. *Main results.* The velocity varies with pore diameter and porosity, with smaller pores yielding faster velocities than larger pores at the same porosity. At 25% porosity and 500 kHz, group velocity ranges from 3147 to 2211 m s^−1^ and phase velocity from 3168 to 2345 m s^−1^ across 0.1–0.6 mm pore diameters. The steady state phase depends on the pore diameter and frequency, with the variation across the measurement plane broadening as both increase, indicating dependence on the microstructure’s pore distribution. *Significance.* The results indicate that the velocity relationship to CT HUs is ill-determined due to the unresolved microstructure. The variation in group velocity impacts pulsed sources, such as those used for histotripsy, while variation in phase velocity affects quasi-continuous sources, including those used for neuromodulation and thermal ablation. Our results emphasize the need to account for the skull microstructure for safer and more effective transcranial focusing.

## 1. Introduction

Transcranial focused ultrasound (tcFUS) is a promising noninvasive modality for treating neurological disorders. Ultrasound can interact with brain tissue through several mechanisms, including thermal and mechanical ablation, neuromodulation, and blood–brain barrier opening. Clinically, ablative tcFUS can treat movement disorders (Elias *et al* 2013), neuromodulation is being investigated for psychiatric disorders (Darmani *et al* 2022), and blood–brain barrier opening may enhance drug delivery to brain tumors (Karmur *et al* 2020). The safety and efficacy of these therapies depend on precisely delivering ultrasound through the skull to the target within the brain.

The heterogeneous patient-specific skull bone aberrates the ultrasound beam, reducing the focusing precision of tcFUS (Clement and Hynynen 2002b). These distortions primarily arise from variations in the speed of sound, but ultrasound propagation also depends on attenuation, including both absorption and scattering (Aubry *et al* 2003). Because skull bones have acoustic properties that differ substantially from soft tissues, significant beam distortions occur during transmission. To compensate for these aberrations, multi-element arrays or acoustic lenses are used to adjust the phase and amplitude of the emitted ultrasound field (Clement and Hynynen 2002a, Daniel *et al* 2024). Alternatively, for single-element transducers, position adjustments with neuro-navigation and amplitude compensation can be used to optimize targeting (Legon *et al* 2018, Pouliopoulos *et al* 2020).

Simulation is an essential tool for predicting and correcting the aberrations induced by the skull (Angla *et al* 2023). However, for ablative procedures, final targeting often relies on patient response and magnetic resonance thermometry to further refine the focal location and intensity (Krishna *et al* 2023). In experimental settings, hydrophone-based corrections have been shown to restore the focal intensity better than current simulation-based corrections, though such measurements are not feasible *in vivo* (Webb *et al* 2018, 2022, Leung *et al* 2022). These observations emphasize the need for improved simulation-based corrections.

One likely source of error within simulations is the reliance on computed tomograpy (CT)-based acoustic property maps derived from Hounsfield units (HUs) obtained from pretreatment CT images. This approach may be limited by the nominal clinical CT voxel size of 0.5 mm isotropic, which cannot resolve the skull microstructure. Several empirical relationships have been proposed to relate acoustic properties to HUs, yet disagreements exist about which relationship is most accurate clinically (Leung *et al* 2019). The velocity relationship is often assumed to be linear, based on a homogeneous fluid model that may not be applicable to heterogeneous skull bones (Rayleigh 1877, Carter and Hayes 1977, Aubry *et al* 2003). In contrast, quantitative ultrasound (qUS) studies have shown that the velocity in bone can depend not only on porosity but also on microstructural features (Grimal and Laugier 2022), suggesting that these simplified assumptions may overlook important contributors to phase aberration.

In this study, we apply qUS approaches to explore whether skull microstructure influences acoustic velocity. Previous qUS simulation studies used digital phantoms designed to represent load-bearing bones such as the femur or calcaneus (Bossy *et al* 2005, Haïat *et al* 2007), where marrow pores are typically modeled as cylinders aligned with the load-bearing direction (Wear 2008). In contrast, skull bone is non-load bearing and lacks oriented cylindrical pores (Larsson *et al* 2014, Alexander *et al* 2019). To better represent skull microstructure, we constructed digital phantoms consisting of spherical marrow pores of a single diameter randomly distributed in a cortical background. Each phantom was assigned a uniform porosity. As such, our phantoms do not model the three-layer macrostructure of skull bone formed by the inner and outer cortical tables and the middle trabecular layer (Brookes and Revell 1998). Instead, they focus on the acoustic properties of bone with a single porosity and pore size. The range of porosities spans both cortical and trabecular bone compositions, with trabecular bone porosities exceeding 30% (Zioupos *et al* 2008).

We evaluated three measures of the microstructure’s effect: group velocity (*V*_g_), phase velocity (*V*_P_), and steady-state phase (*ϕ*_s_), each relevant to different applications of tcFUS. Group velocity describes the propagation of the broadband pulses used in histotripsy treatments (Sukovich *et al* 2019), while phase velocity describes the propagation of single-frequency continuous waves used in most other mechanisms of tcFUS (Elias *et al* 2013, Darmani *et al* 2022). The steady-state phase directly impacts the phase correction of multi-element arrays (Flax and O’Donnel 1988, Wang *et al* 2024). Across all measures, we find that microstructural variations affect ultrasound propagation.

## 2. Methods

We conducted full-wave ultrasound simulations through digital phantoms using the k-Wave software package to test how the microstructure affects the composite velocity. Specifically, we constructed digital phantoms at several target porosities (2.5%–80%) and with fine-to-course microstructure pore size (0.1–0.6 mm). Using these phantoms, we simulated a through-transmission experiment to determine *V*_g_ and *V*_P_ as a function of porosity and pore size. As hypothesized, we find that, for both group and phase velocity, the relationship between porosity and speed of sound depends on the underlying microstructure that cannot be resolved with typical clinical CT.

### 2.1. Bone phantoms

**Figure 1.**
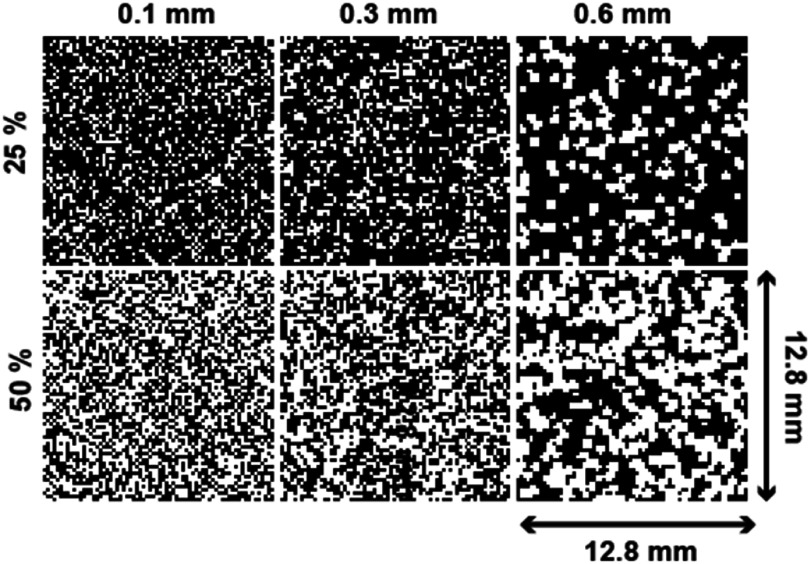
Phantom central transverse cross sections for pore diameters of 0.1 mm (left), 0.3 mm (middle), and 0.6 mm (right) at nominal 25% (top) and 50% (bottom) porosities. Black indicates cortical bone, while white represents marrow.

Three-dimensional bone-mimicking phantoms (12.8 × 12.8 × 8 mm; isotropic 0.05 mm resolution) consisting of various porosities and microstructures were generated. Each phantom consisted of spherical red marrow pores of a single pore diameter randomly placed into a cortical background, allowing overlapping pores. The acoustic properties of the constituent materials and nominal values for water are provided in table 1. Five pore diameters (0.1 mm, 0.2 mm, 0.3 mm, 0.4 mm, 0.6 mm) smaller than the smallest wavelength used in this study (1.45 mm in marrow and 3.5 mm in bone at 1 MHz) were chosen. The pores were allowed to overlap to better mimic the complex trabecular bone microstructure. Each pore diameter was employed in 11 phantoms, ranging in porosities from 2.5% to 80%, for a total of 77 phantoms. Figure 1 shows cross sections through 25% and 50% porosity phantoms for three pore diameters. Details of the phantoms’ digital construction are provided in the supplemental materials.

**Table 1.** Acoustic properties of constituent materials (Hasgall *et al* 2022) and water.

Material	Speed of sound (m s^−1^)	Attenuation (Np m^−1^)	Density (kg m^−3^)
Water	1500	0	1000
Red marrow	1450	12.55	1029
Cortical bone	3514	54.55	1908

### 2.2. 3D simulations

All simulations were completed using k-Wave version 1.4, a well validated full-wave pseudo-spectral time-domain solver (Treeby and Cox 2010a, Aubry *et al* 2022). The extent of the grid was 12.8 × 12.8 × 10.8 mm with an isotropic spacing of 0.05 mm. A 20 voxel-thick perfectly matched layer with 2.0 Np/voxel attenuation was added externally to the end plane to achieve an effective infinite domain. Transverse matching layers were unnecessary as the transverse periodicity causes the uniform source to remain uniform as it propagates (i.e. a plane wave as described in the k-Wave MATLAB examples) (Treeby *et al* 2014).

**Figure 2.**
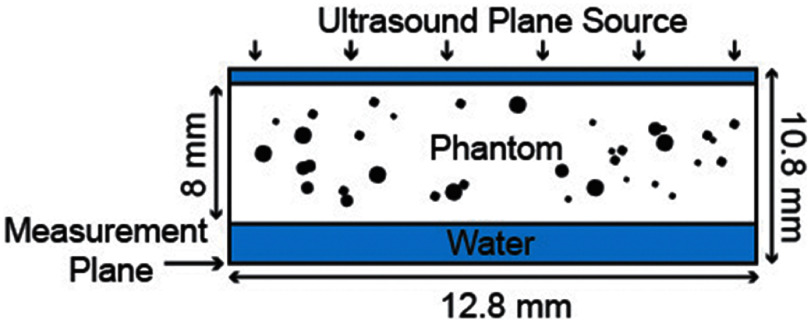
Longitudinal slice of a typical phantom, with the transverse source plane at the top propagating a single cycle or continuous plane wave through the phantom to the transverse measurement plane at the bottom. Note that the pores appear to have different diameters; however, this results from taking a 2D slice through 3D spherical pores with the same diameter.

Each source was generated with one of four center frequencies (250 kHz, 500 kHz, 750 kHz, and 1 MHz) covering the range commonly found in therapeutic ultrasound applications. Single-cycle pulsed plane wave sources were used to find *V*_g_ and *V*_P_. In addition, a continuous plane wave source was used to find ∅_s_. The pressure sources were propagated in the *z*-direction from the front plane of the grid. The 8 mm thick bone phantoms were placed ten voxels into the grid, resulting in water layers of 0.5 mm in front and 2.3 mm behind the phantom, as shown in figure 2. (The pores shown in figure 2 appear to have different diameters; however, this results from taking a 2D slice through 3D spherical pores with the same diameter.) The time-varying pressure was recorded in the last transverse plane (i.e. in water after propagating through the phantom). The source pressure was smoothed to reduce high spatial frequency components. Filtering reduces numerical errors due to source discretization, which can cause non-physical oscillations. Uniform sources are not strongly affected by this filtering. The medium properties, including speed of sound, attenuation, and density, were also smoothed, decreasing errors in the phase’s numerical propagation (Tabei *et al* 2002). Smoothing minimizes Gibbs phenomena, staircasing effects, and aliasing, leading to more accurate simulations (Tabei *et al* 2002, Robertson *et al* 2014, 2017a). The default Blackman window in k-Wave was used for all smoothing in this study. The Blackman filter is used by default because it better reduces oscillations (low side lobes) compared to Hanning or Hamming windows; however, it also has a wider main lobe, which results in more smoothing (Treeby and Cox 2011). The reference speed of sound was set to the minimum (marrow, 1450 m s^−1^), which reduces non-physical numerical dispersion but requires a more stringent stability criterion in the time step (Treeby *et al* 2016).

The time step was then defined using a specified Courant–Friedrichs–Lewy (CFL) condition, where a smaller CFL condition results in a smaller time step (Tabei *et al* 2002). The time step must be reduced with decreasing frequency to ensure stability; as such, the CFL was set to 0.024, 0.035, 0.06, and 0.08 for source frequencies 250 kHz, 500 kHz, 750 kHz, and 1 MHz, respectively. The single pulse source simulation duration was set to 20 *μ*s, sufficient to include the arrival of the pulse through every phantom. The continuous source simulation duration was set to 80 *μ*s, which reaches a steady state in all cases, as determined by convergence testing explained next.

Convergence testing ensures that numerical solutions remain stable and accurate as the discretization of parameters (e.g. spatial and temporal resolution) is refined. It typically involves demonstrating that smaller discretization results in negligible changes to the solution, indicating numerical stability and accuracy (Robertson *et al* 2017a). Convergence testing related to the time step size at all frequencies by decreasing the initial CFLs by a factor of 2. We used the smallest and largest pore diameter phantoms at 25% and 50% nominal porosity for testing, as shown in figure 1. The average percentage difference in the group or phase velocity measurements was used as a convergence metric. The largest percentage difference in the group and phase velocities was 0.064%, occurring for the phase velocity in the 0.6 mm pore, 50% porosity phantom at a frequency of 1 MHz. All convergence tests resulted in less than a tenth of a percent difference. The convergence results for each test are included in supplemental table 1. We did not repeat the time step convergence test with the continuous source, as its stability and convergence can be inferred from the pulsed source convergence test.

Convergence testing for the length of the time duration was not necessary for the pulsed source, as the duration was long enough for the initial envelope to arrive in all cases. Convergence testing for the time duration with continuous sources was completed by doubling the duration to 160 *μ*s with the 250 kHz source, chosen because it has the longest wavelength. We used the smallest and largest pore diameter phantoms at 25% and 50% nominal porosity for testing. The percentage difference in ∅_s_ across the measurement plane was used as a metric for convergence. This metric had a maximum 0.03% difference, occurring for the 0.6 mm, 50% porosity phantom, indicating the simulation reaches a steady state in 80 *μ*s. We did not conduct convergence testing related to the grid spacing, as our grid spacing results in 29 points per wavelength in the material with the minimum velocity at 1 MHz, which is well above the recommended minimum of 4 points per wavelength (Robertson *et al* 2014).

The simulations included acoustic absorption, which in k-Wave is modeled by a frequency power law that relates two loss terms to the frequency-dependent absorption (Treeby and Cox 2010b). These terms are the power law pre-factor and the frequency exponent. The exponent is related to the dispersion as the Kramers–Kronig (k–k) relationship requires (Treeby and Cox 2010b). k-Wave derives a power law absorption relationship using a similar approach to the band-limited k–k relationship (Waters and Hoffmeister 2005, Treeby and Cox 2010b). We assumed the materials were dispersionless to focus on dispersion due to inter-voxel path length changes. The dispersion introduced by the power law absorption can be eliminated by setting the frequency exponent to 2. This means the constituent materials are non-dispersive, although dispersion may occur due to interactions within the microstructure. A linear power law relationship can still be defined at one frequency by dividing the pre-factor by that frequency in units of MHz (assuming the pre-factor is defined at 1 MHz).

### 2.3. Group and phase velocity measurement

A 3D matrix of received signals at the measurement plane, with the row and columns corresponding to the transverse extents of the grid and with time in the third dimension, was acquired. A single reference signal at each frequency was found, as the water-only pressure is spatially uniform for a plane wave, as confirmed by simulation. The group and phase velocities were found over the measurement plane for each acquired signal by the substitution method (Strelitzki *et al* 1996) using the following equation:

\begin{align*}V = \frac{{{C_{\text{w}}}}}{{1 + \frac{{{C_{\text{w}}}\Delta t}}{d}}}\end{align*}

where *C*_w_ is the speed of sound in water, *d* is the phantom thickness, and Δ*t* is the time difference (defined below) corresponding to either the group or phase velocity.

Δ*t* is determined by different methods for group or phase velocity. For group velocity, *V*_g_ was calculated by finding Δ*t* from the normalized cross-correlation of the envelopes in the measurement plane (obtained from the absolute value of the Hilbert transform of each signal) compared to the envelope of the water-only reference. The time lag corresponding to the peak value of the cross-correlation function defines Δ*t*.

For phase velocity, *V*_P_ was calculated by taking the Fourier transform of each signal and a water-only reference. The time-domain signal was zero-padded to four times its initial length (20 *μ*s to 80 *μ*s) to increase the frequency resolution. The phase at the carrier frequency was determined from the imaginary and real parts of the data as arctan(imag/real). Next, we found the phase difference in the Fourier domain by complex conjugation between the signal and reference phases. Finally, we performed phase unwrapping by computing the cumulative sum of the phase increments between adjacent Fourier coefficients, beginning with the Fourier coefficient adjacent to the DC phase. The unwrapped phase is related to Δ*t* by the following equation:

\begin{align*}\Delta t = \frac{{\Delta \varphi \left(f\right)}}{{2\pi f}}.\end{align*}

For continuous sources, the steady-state phase *ϕ*_s_ observed on the measurement plane can be found in the time domain. After 90% of the simulation duration (72 *μ*s), we found the first peak in each signal and a water-only reference. The time difference between these peaks can be related to the steady state phase difference by the angular frequency. The distribution of phases over the measurement plane was analyzed using a phase histogram.

To investigate the effect of microstructure on the group and phase velocities within different bone phantom configurations, we conducted simulation experiments on each of our 77 phantoms. These experiments explored the relationship of *V*_g_ and *V*_P_ to porosity, pore size, and frequency. Finally, we related *ϕ*_s_ to pore size, pore distribution, and frequency.

## 3. Results

**Figure 3.**
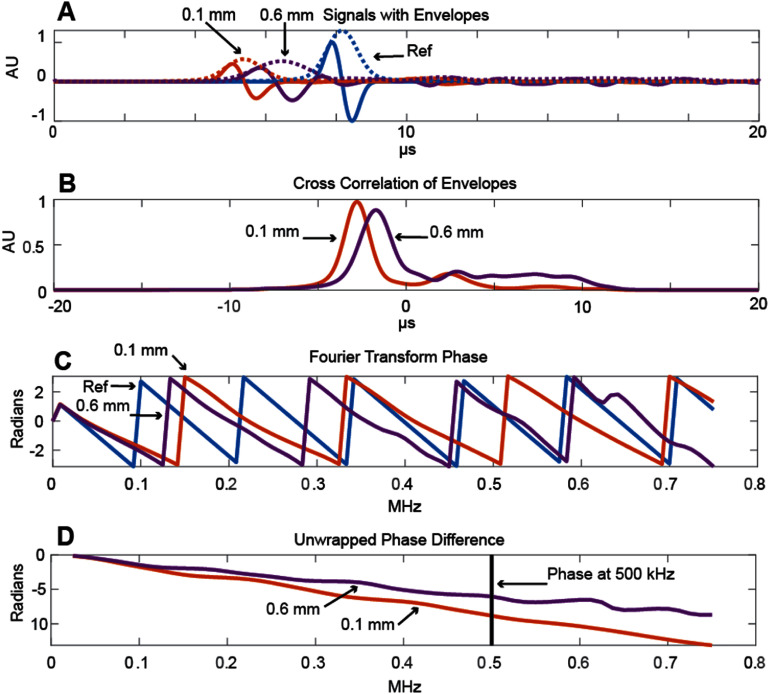
Example signals and signal processing methods to find the group and phase velocities. Plot (A) shows the 500 kHz pulsed time domain signals through the 0.1 mm and 0.6 mm pore diameter 25% porosity phantoms. A water-only reference signal is included. The dotted lines correspond to the envelope of each signal. Plot (B) shows the normalized cross-correlation of the 0.1 mm and 0.6 mm pore signal envelopes with the reference envelope, from which the group velocity is calculated. Plot (C) shows the frequency-dependent wrapped phase for each signal, calculated using the Fourier transform. Plot (D) shows the phase difference results after unwrapping.

Figure 3 provides an example of the signal measured through nominally 25% porosity phantoms with 0.1 mm or 0.6 mm pore diameters. The ultrasound source is a planar single-cycle pulse centered on 500 kHz. Plot (A) shows the signals at the center of the measurement plane and a water-only reference signal (which is independent of position since the source is a plane wave). Solid lines show the signals and the dotted lines show the envelopes of each signal. Plot (B) shows the normalized cross-correlation of the envelopes with the reference envelope. The peaks correspond to the time lag between the two envelopes: −2.79 *μ*s for the 0.1 mm pore phantom and −1.72 *μ*s for the 0.6 mm pore phantom. These lags result in a *V*_g_ of 3147 m s^−1^ for the 0.1 mm pore phantom and 2211 m s^−1^ for the 0.6 mm phantom (equation (1)). The peak normalized cross correlation value quantifies the wave’s envelope distortion and is lower for the 0.6 mm pore phantom (0.88 AU) compared to the 0.1 mm pore phantom (0.98 AU).

Plot (C) shows the calculation of *V*_P_, beginning with the frequency-dependent wrapped phase for each signal from 0 to 750 kHz calculated from the angle of the Fourier transform coefficients. Plot (D) shows an unwrapped phase. *V*_P_ can be calculated from the unwrapped phase difference at the center frequency (500 kHz) using equations (2) and (1). In this example, the *V*_P_ is 3168 m s^−1^ for the 0.1 mm pore phantom and 2345 m s^−1^ for the 0.6 mm phantom.

**Figure 4.**
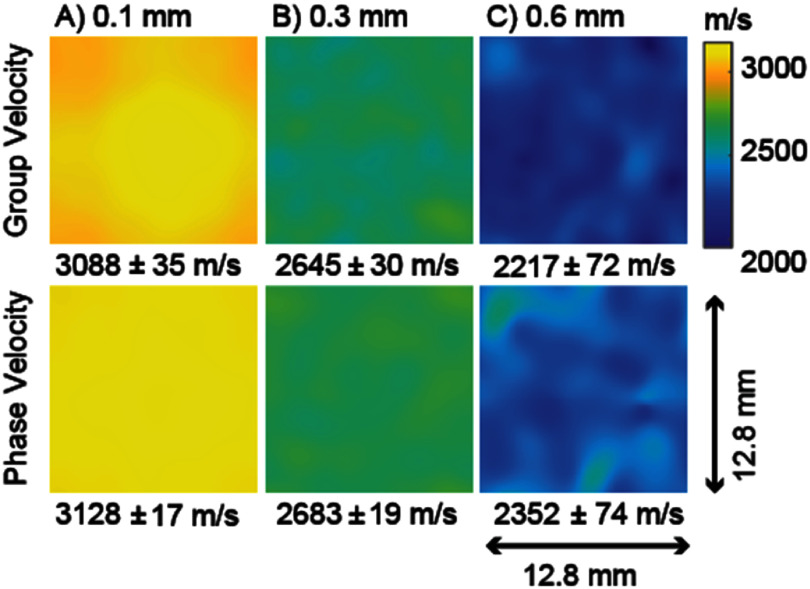
Example group and phase velocities at 500 kHz in the measurement plane through 0.1 mm (A), 0.3 mm (B), and 0.6 mm (C) pore diameter 25% porosity phantoms. The top row shows the group velocity and the bottom row shows the phase velocity. The average and standard deviation of the velocities across the measurement plane are reported below each image.

Figure 4 shows example images of *V*_g_ and *V*_P_ over the measurement plane through 0.1 mm (A), 0.3 mm (B), and 0.6 mm (C) pore, 25% porosity phantoms at 500 kHz. The top row shows the *V*_g_ images, and the bottom row shows the *V*_P_ images. All images use the color scale shown on the right. Below each image, the mean velocity and the standard deviation of velocity over the measurement plane are included. *V*_g_ and *V*_P_ both decrease with increasing pore size. *V*_g_ is slower than the *V*_P_ in each case, suggesting negative dispersion. The variation in velocity across the measurement plane generally increases with pore size, with the 0.1 mm pore velocities appearing more uniform and the 0.6 mm pore velocities having more spatial variation. The increase in standard deviation quantifies this increasing spatial variation with pore size.

**Figure 5.**
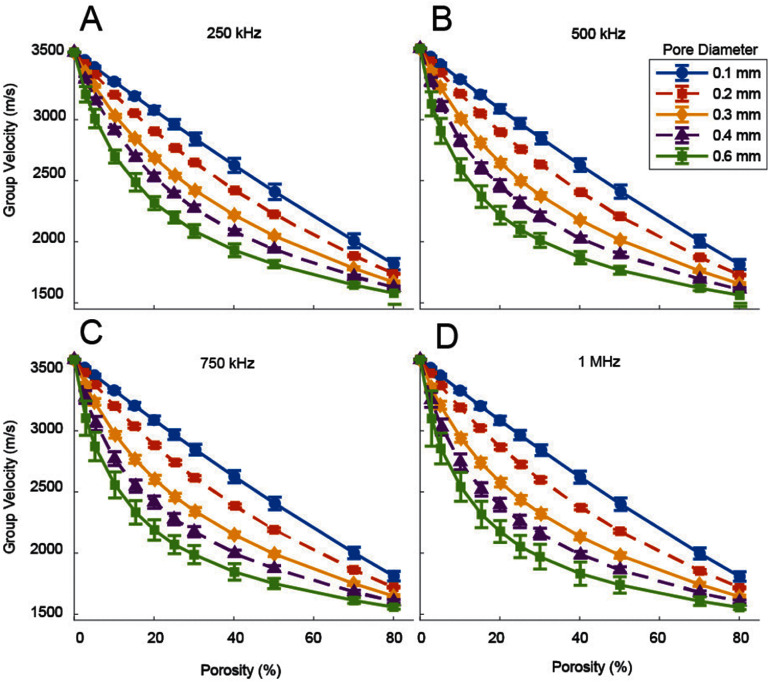
Group velocity as a function of porosity. Each line represents a different pore diameter, ranging from 0.1 mm to 0.6 mm. Four frequencies are shown (top left = 250 kHz, top right = 500 kHz, bottom left = 750 kHz, bottom right = 1 MHz). The error bars represent the standard deviation across the measurement plane.

Figure 5 shows the mean and standard deviation of *V*_g_ as a function of pore size and porosity at the four frequencies (*A* = 250 kHz, *B* = 500 kHz, *C* = 750 kHz, *D* = 1 MHz). The pore diameter legend in the top right applies to all frames. *V*_g_ decreases with porosity for all pore sizes. Smaller pore diameter phantoms have faster group velocities than larger pore diameter phantoms for a given porosity. The smaller pore diameter curves are more linear than the larger ones, which become increasingly concave with pore size. The *V*_g_ curves converge to the values expected in bone-only phantoms (3514 m s^−1^) and towards the values expected for marrow-only (1450 m s^−1^) phantoms at 100% porosity.

The error bars in figure 5 represent the standard deviation across the measurement plane. The error bars are smaller than the separation between the plots and are sometimes obscured by the data markers. The variation increases with increasing pore size in medium to large pore phantoms, as indicated by larger error bars. In these cases, the error bars generally also increase with increasing frequency. For example, the 0.6 mm pore, 25% porosity phantom has a *V*_g_ of 2198 ± 45 m s^−1^ at 250 kHz compared to 2053 ± 91 m s^−1^ at 1 MHz. An additional figure showing the relationship of measured *V*_g_ to frequency is included in supplementary figure 1.

**Figure 6.**
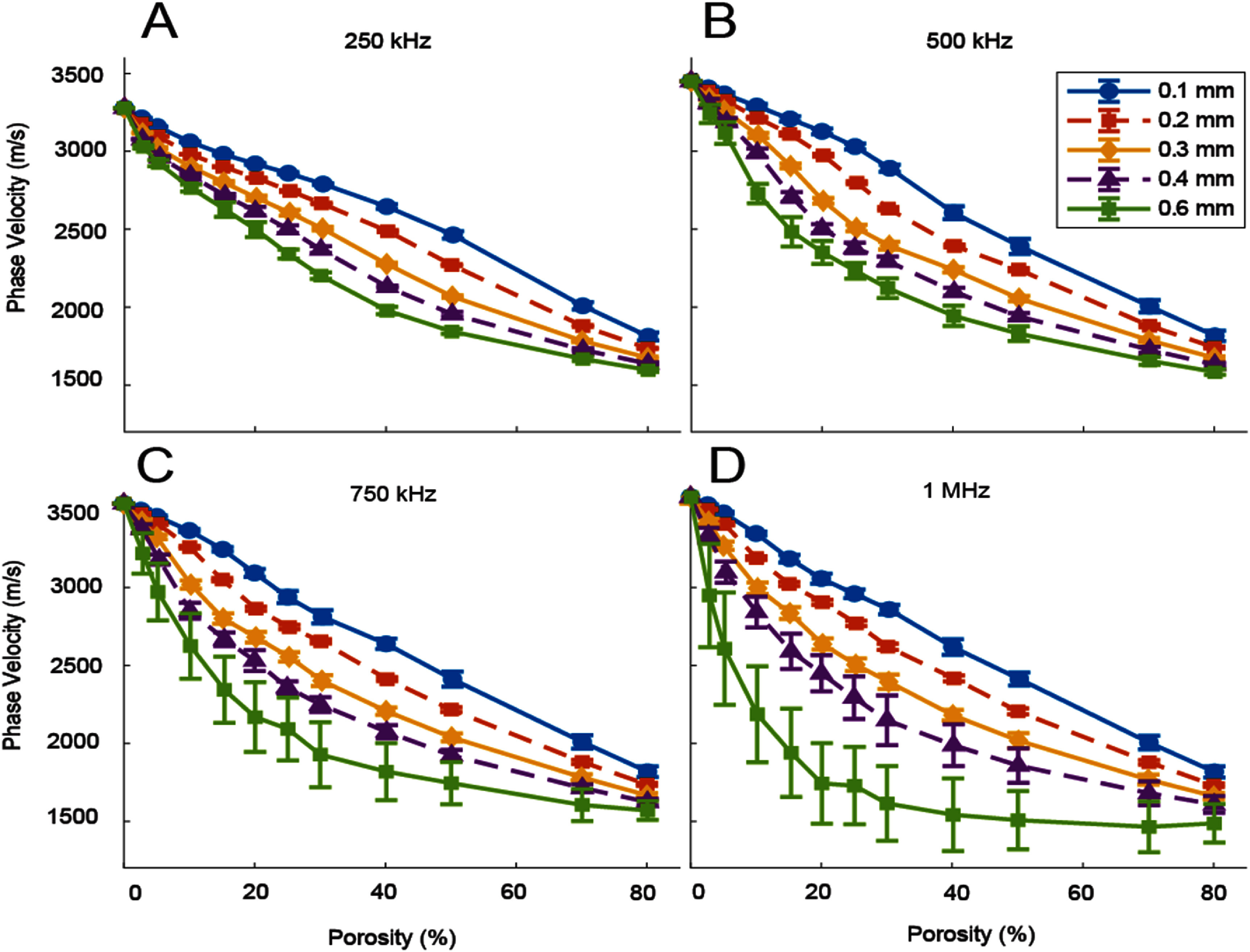
Phase velocity as a function of porosity. Each line represents a different pore diameter, ranging from 0.1 mm to 0.6 mm. Four frequencies are shown (*A* = 250 kHz, *B* = 500 kHz, *C* = 750 kHz, *D* = 1 MHz). The error bars represent the standard deviation across the measurement plane.

Figure 6 shows *V*_P_ as a function of pore size and porosity at four frequencies (250 kHz, 500 kHz, 750 kHz, and 1 MHz). The legend indicates each curve’s pore diameter and applies to all frames. Similar to *V*_g_, *V*_P_ decreases with increasing porosity. At a single porosity, *V*_P_ is slower through larger pore-size phantoms. The smaller pore diameter curves are more linear than the larger ones, with the nonlinearity increasing with pore diameter and frequency. The nonlinearity of the *V*_P_ curves increases more noticeably with frequency compared to the *V*_g_ curves shown in figure 5. At 1 MHz, the 0.6 mm pore curve rapidly decreases from 3582 to 1614 m s^−1^ over a 0% to 30% porosity range. This rapid decrease can be explained by the sensitivity of the measurement method to multi-path scattering, as described in the discussion. An additional figure showing the relationship of measured *V*_P_ to frequency is included in supplemental figure 2.

The error bars represent the standard deviation across the measurement plane. In most cases, the error bars are smaller than the separation between lines, with some error bars obscured by the data marker. The error bars generally increase with pore size and frequency. For example, the 25% 0.6 mm phantom has a *V*_P_ of 2341 ± 29 m s^−1^ at 250 kHz compared to 2093 ± 202 m s^−1^ at 750 kHz.

Notably, at 0% porosity, *V*_P_ converges to values different than the expected value for the bone-only phantom (3514 m s^−1^). We measured phase velocity in a bone-only phantom and found the following values: 3280 m s^−1^, 3451 m s^−1^, 3540 m s^−1^, and 3583 m s^−1^ at 250 kHz, 500 kHz, 750 kHz, and 1 MHz, respectively. This variation with frequency may be explained by our particular method of phase velocity measurement, which includes some effects of reflections, as described in the discussion section. The phase velocity converges towards the value at 100% porosity expected for marrow-only (1450 m s^−1^) phantoms.

**Figure 7.**
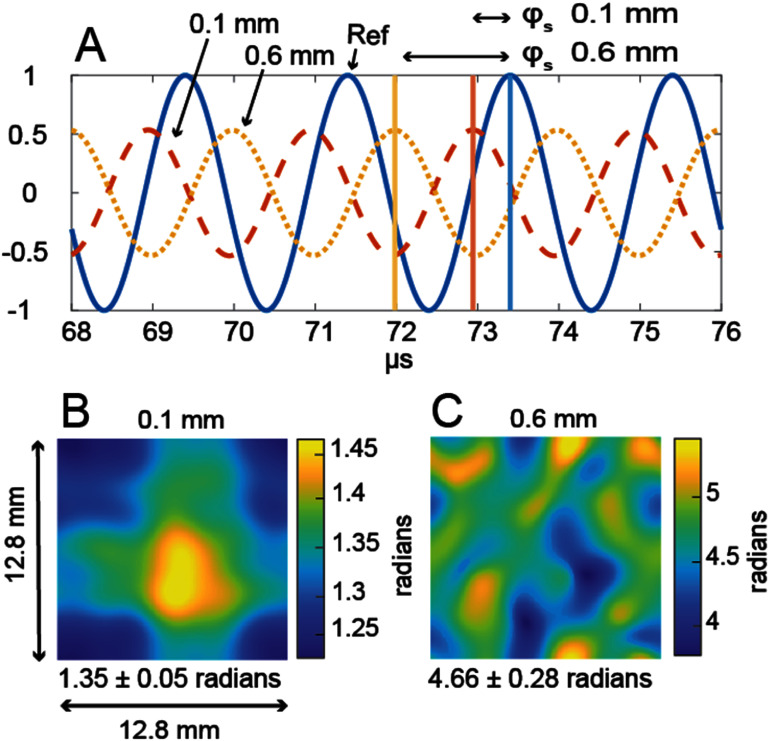
Example steady-state phase calculation over the measurement plane in two representative phantoms at 500 kHz. Row (A) shows the steady-state time signals through 0.1 mm and 0.6 mm 25% porosity phantoms and the water-only reference. The relative phase difference is found from the time difference between peaks (vertical lines). The bottom row shows the phase difference across the measurement plane for the 0.1 mm (B) and 0.6 mm (C) pore phantoms. The mean phase and standard deviation across the measurement plane are reported below each image.

Many focused ultrasound treatments use quasi-continuous wave sources. We therefore employed simulations looking at the steady-state phase in the measurement plane for varying pore size and porosity phantoms with a quasi-continuous source. Figure 7 shows examples of steady-state signals and the signal processing method used to find a relative phase. Examples include signals through 0.1 mm and 0.6 mm pore sizes with 25% porosity phantoms and a water-only reference for a 500 kHz quasi-continuous planar source. The time signals are shown in figure 7(A) over a limited interval from 68 *μ*s to 76 *μ*s to improve visibility. The algorithm finds the first peak after 90% of the signal has arrived (72 *μ*s), as indicated by the vertical lines. Then the relative phase difference in radians is found from the time difference between this peak and the reference peak after considering the 2 *μ*s period. The lower images show the phase across the measurement plane after transmission through the 0.1 mm (B) and 0.6 mm (C) pore size 25% porosity phantoms. The phase is approximately uniform through the 0.1 mm pore size phantom but has some spatial variation through the 0.6 mm pore size phantom. The average phase within the measurement plane ranges from 1.35 to 4.66 radians over a pore diameter range of 0.1–0.6 mm, illustrating that the phase depends on the pore diameter.

**Figure 8.**
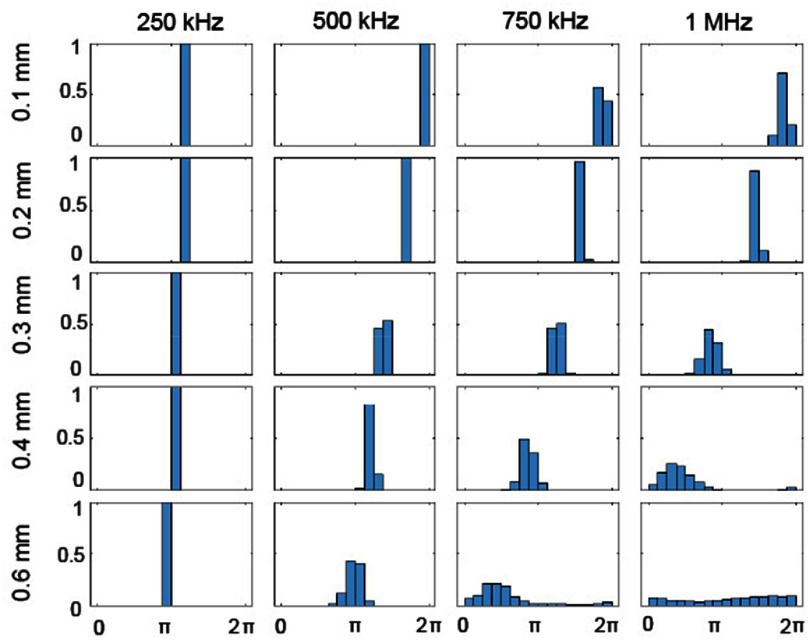
Steady-state phase histograms over the measurement plane for 25% porosity phantoms. The columns correspond to four frequencies while the rows correspond to five pore diameters. The bin size is one-sixteenth of a period (2*π*/16).

Figure 8 shows a histogram of *ϕ*_s_ for all pore diameters and frequencies at 25% porosity. The histogram’s intensity represents the probability of a pixel falling within the radian value covered by each bin, 2*π*/16. This bin size corresponds to one-sixteenth of a period. We chose this bin size such that the phase variation as a function of position can be evaluated with the Goodman criterion, which corresponds to two bins (2*π*/8). The Goodman criterion represents the precision in phase needed for good focusing (Goodman 1985, Aubry *et al* 2003). The columns of the figure correspond to four frequencies, while the rows correspond to five pore diameters. The phase at 250 kHz depends slightly on the pore size, shifting 0.95 radians from an average of 3.87 radians to 2.92 radians between the 0.1 mm to 0.6 mm pore diameters. The phase at 250 kHz falls into a single bin for all pore sizes. The phase at 500 kHz shifts with pore size, shifting from an average of 6.05 radians to 3.08 radians between the 0.1 mm to 0.6 mm pore diameters. The phase histogram also broadens with increasing pore size, starting with a single bin in the 0.1 mm and 0.2 mm rows and finishing with four bins in the 0.6 mm row. The two trends that the average phase shifts and the phase histogram broadens with increasing pore diameter, as observed at 500 kHz, become even more prominent at 750 kHz and 1 MHz, as seen in figure 8. Phase offsets, constant across pore sizes, were applied to the phases within each frequency column to avoid phase wrapping effects; as such, the relative phase values should not be compared across frequencies.

## 4. Discussion

Our study provides evidence that the acoustic property maps used for simulation-based phase correction are sensitive to skull microstructure unresolved by typical clinical CT. Variations in group and phase velocities arising from microstructural heterogeneity within a clinical CT voxel introduce uncertainty that can reduce the precision of transcranial ultrasound focusing. The variation in group velocity mainly impacts short pulses, such as those used for histotripsy, while variation in phase velocity affects quasi-continuous waves, such as those used for thermal ablation procedures. This study is related to a companion study, which showed that unresolved microstructure influences the acoustic intensity loss through digital phantoms similar to those used here (Clinard *et al* 2025).

Across all metrics, microstructural effects increase with increasing pore size and frequency. Figures 5 and 6 show that higher frequencies produce greater sensitivity of velocity to pore size. Figure 8 shows that *ϕ*_s_ broadens with increasing frequency and pore size. At 250 kHz, *ϕ*_s_ is largely independent of pore size within the range of pore sizes simulated, whereas ultrasound at higher frequencies exhibits greater phase shifts. This frequency/pore size dependence is accompanied by greater spatial variability. One mechanism underlying this frequency dependence is scattering, which can lead to negative dispersion as observed in supplemental figure 2 (Haïat *et al* 2008). Increased scattering may result in slower velocities at higher frequencies due to longer path lengths. Negative dispersion is sometimes termed ‘anomalous’ because it contradicts the prediction of logarithmic positive dispersion based on k–k relationships (Marutyan *et al* 2006). These trends indicate that the size and spatial arrangement of microstructural heterogeneities can influence ultrasound propagation, particularly when using higher-frequency sources.

Most prior empirical velocity–porosity relationships assume linearity based on a homogeneous fluid model (Carter and Hayes 1977, Aubry *et al* 2003). Our results indicate that microstructural heterogeneities can introduce nonlinear effects, suggesting a quadratic term may be needed, especially for coarser heterogeneities. Heterogeneous ultrasound theories (e.g. multiple scattering) predict the nonlinear trends with porosity, pore-size, and frequency we observe, but their simplifying assumptions break down across our phantoms’ broad variability in porosity, size, and shape (Foldy 1945, Waterman and Truell 1961, Sehgal and Greenleaf 1984). Similarly, real skull bones have diverse microstructures, suggesting no single theory can fully describe transmission (Alexander *et al* 2019). Nonetheless, our results are consistent with theory and experiments showing complex microstructural interactions influence both group and phase velocities (Haïat and Naili 2011, Haïat *et al* 2008, Bossy *et al* 2005, Mézière *et al* 2014).

### 4.1. Clinical implications of microstructural velocity variations

Our results suggest that microstructure can reduce the focal quality through effects that current simulation techniques cannot predict. The Goodman criterion estimates that a phase precision of 2*π*/8 is required for adequate focusing of phase arrays and is related to Goodman’s original observation that the focal quality degrades from a diffraction-limited case when the root mean square phase error is around 1 radian (Goodman 1985). For focused ultrasound arrays, this criterion indicates that the array restores 80% of the ideal focal energy (Aubry *et al* 2003). Clinically, the phase error is introduced when performing simulation-based phase aberration correction, typically with time-reversal simulations, where the phase error accumulates in the propagation direction (Marquet *et al* 2009). Figure 8 shows that increasing frequency and pore size lead to phase variations exceeding the Goodman criterion, suggesting that microstructure-induced variations in velocity can be sufficient to affect multi-element array focusing at clinically relevant frequencies.

Previous studies of simulation guidance for both single-element and multi-element arrays have reported errors in focal position, volume, and intensity compared to hydrophone scans (Leung *et al* 2021, Krokhmal *et al* 2025). This suggests the velocity relationship to HUs can improve, although there is also corresponding uncertainty in skull attenuation, explained in part by microstructural variations (Clinard *et al* 2025). However, uncertainty in velocity variations does not explain all the variation in previous studies (Leung *et al* 2019), as at least one study found increased error in focal position at both low and high frequencies compared to mid-range frequencies (Krokhmal *et al* 2025). Another study explored the sensitivity to sound speed variation with single-element transducers, and found the variation must be kept below 4%, 11%, and 9% to reduce errors to 5% (focal pressure), 20% (focal volume), and 1.5 mm (focal position), respectively (Robertson *et al* 2017b). We note the velocity variations found here and reported in the literature are much larger than these requirements.

### 4.2. Strategies for mitigating microstructure-induced errors

High-resolution imaging may reduce velocity variations that arise from sub-voxel microstructure, but even the highest typical clinical CT or MRI resolution (0.5 mm to 1 mm) obscures heterogeneities that are not represented by an average velocity. This is evident from figure 6, where error bars grow with larger pore sizes and increasing frequency, and within figure 8, where the steady-state phase broadens. At 1 MHz, the phase variation meets the Goodman criterion for pore sizes ⩽0.2 mm, suggesting this is the target resolution to minimize microstructural uncertainty. As a possible solution, it has been clinically shown that deep learning approaches can enhance CT’s effective spatial resolution (Rytky *et al* 2024). Alternatively, photon-counting CT can achieve a nominal isotropic resolution of 0.2 mm, although other challenges have limited its broader adoption (Wehrse *et al* 2019, Benson *et al* 2022, Rajagopal *et al* 2024).

In the absence of increased resolution, a machine learning characterization of the sub-voxel microstructure based on clinical CT or qUS may inform velocity relationships without explicitly resolving the microstructure (Mohanty *et al* 2019, White *et al* 2021). For example, it may be possible to determine which patients or skull regions have coarser microstructures, which may be better represented with nonlinear velocity relationships.

The limitations in simulation guidance motivate the development of feedback mechanisms to confirm and refine the focal position, as is done in thermal applications of tcFUS (Odéen and Parker 2019). Acoustic radiation force imaging is a promising method for measuring the focal location and was recently demonstrated in humans (Mohammadjavadi *et al* 2025, Odéen *et al* 2025). In addition, performing phase correction based on MR-Hydrophone measurements may be possible (Passe-Carlus *et al* 2025).

### 4.3. Influence of measurement methods on velocity estimates

It can be noted that this study relies to some degree on our particular group and phase velocity measurements, which are sensitive to the measurement method employed (Nicholson *et al* 1996). A key challenge in velocity estimation is that the substitution method (equation (1)) assumes a single, coherent transmission pathway—commonly called a ballistic wave assumption. A ballistic wave is a direct, coherent wavefront that propagates through a medium without significant scattering or reflections (Bossy *et al* 2005). In heterogeneous media, scattering introduces attenuation, dispersion, and wave distortions, complicating both time and frequency domain velocity calculations (Chen and Chen 2006, Wear 2020). Group velocity is typically determined in the time domain. In this study, we used a cross-correlation method (figure 3(B)), which is robust to waveform distortions, requires no user input, and considers the entire pulse. Alternative time domain approaches, such as zero crossings, are more susceptible to waveform distortions and were therefore not used here.

Phase velocity was calculated in the frequency domain using a fully automated method that analyzes the entire acquired signal from a short, broadband ultrasound pulse, enabling a simple unwrapping routine without user input. In contrast, prior approaches required a user-defined time window to isolate the ballistic wave (Nicholson *et al* 1996), a process which is difficult to automate and becomes impractical when the ballistic wave overlaps with reflections. By analyzing the entire signal, we assumed that the ballistic component of the wave dominates the phase response. However, this approach still means that some reflections—from both pores and the planar surfaces of the bone phantom—will affect the measured phase velocity. This likely contributes to the rapid decrease in the measured phase velocity observed at high frequencies and larger-pore phantoms, as seen in figure 5. It may also explain the observed frequency dependence of the phase velocity in the bone-only phantoms, as described in results. However, our study relies on comparative trends using a consistent measurement approach across simulations, which adds some robustness. The phase velocity estimation remains a challenging problem in both hydrophone and simulated through transmission measurements, which partly motivated our examination of the steady-state phase as an additional complementary metric of microstructural influence. Although alternative phase velocity estimation techniques—such as zero crossings, phase tracking, and split spectrum processing—have been proposed (Strelitzki and Evans 1996, Nicholson *et al* 2000, Chen and Chen 2006, Lin *et al* 2006), these methods are generally less robust or more challenging to automate reliably.

### 4.4. Study limitations

We note several limitations inherent in our study. Notably, we emphasize that our experiment was designed to elucidate the effects of microstructure as characterized by just two global parameters: porosity and pore size. As such, our digital phantoms employ a simplified representation of skull bone, similar to those used in previous qUS studies (Karbalaeisadegh *et al* 2018, Yousefian *et al* 2018, 2021). Each phantom assumes a uniform porosity and consists of randomly distributed, overlapping, same-sized spherical pores consistent with prior measurements of pore size (Larsson *et al* 2014). This contrasts with the use of non-overlapping cylindrical or circular pores in earlier studies. While our phantoms may better reflect real skull bone, we purposely do not include depth-dependent porosity (Alexander *et al* 2019), variable pore shape and size, distinct cortical and trabecular layers, or anisotropy (Chen *et al* 2013). We note that while both simulated and physical experiments with real skull bones have found velocity variations across and within skulls (Leung *et al* 2019), the microstructural contribution to this variation is uncertain due to the complex heterogeneity. Therefore, while our phantoms do not exactly represent skull bone, their simplifying assumptions are necessary to demonstrate that microstructure affects relative velocities.

Our simulation routine further incorporates several modeling approximations. We applied an attenuation power law factor of 2, resulting in dispersionless materials according to the k–k relationship implemented in k-Wave. This choice allowed us to focus on the effects of microstructure on velocity and dispersion; however, the resulting quadratic attenuation preferentially attenuates the higher frequency components of broadband pulses, which may influence velocity estimation. Nonlinear effects were excluded from our simulations, which is appropriate for pressure amplitudes less than 1 MPa (Aubry *et al* 2022). We employed k-Wave’s fluid model, neglecting viscoelastic interactions and mode conversion, which is a reasonable approximation for normally incident plane waves (Treeby and Saratoon 2015). However, viscoelastic effects may occur at the bone-marrow interfaces as solid and fluid waves are coupled as described by Biot theory (Haire and Langton 1999). The phase velocity can be affected by fast and slow waves, which arise from the relative motion of the marrow moving in phase (fast) or out of phase (slow) with the solid bone (Marutyan *et al* 2006, Kubo *et al* 2011). While the field of therapeutic ultrasound has traditionally primarily used fluid simulations, a recent study indicated elastic simulations are more accurate for oblique angles and shallow focusing (Gao *et al* 2023). Future studies may further explore viscoelastic interactions in skull microstructure and their potential influence on phase and amplitude compensation. While these interactions could modify absolute velocity values, their relative dependence on microstructure would preserve the trends observed in this study.

## 5. Conclusion

This study indicates that a single clinical CT HU can correspond to a variety of group and phase velocities, depending on the skull microstructure that typical clinical CT cannot resolve. This intrinsic variation in velocity may contribute to the variability of relationships reported in the literature. The skull microstructure influences these velocities, with the greatest effect occurring within coarser microstructures and at higher frequencies. The variation in group velocity limits phase correction for broadband pulses, including those used in histotripsy. The variation in phase velocity limits phase correction for quasi-continuous treatments, including neuromodulation and thermal ablation. We demonstrate that the phase error increases within coarser microstructures and at higher frequencies and eventually becomes dependent on the microstructure’s spatial distribution. These findings motivate using lower frequency sources that are less sensitive to microstructure. Phase correction of higher frequency sources may benefit from improved phase velocity relationships to CT HUs, which account for nonlinearities and voxel size dependency. Recognizing the uncertainty in CT-based simulations as a pre-treatment tool can potentially contribute to safer and more effective treatments.

## Data Availability

The data that support the findings of this study are available upon reasonable request from the authors. Supplementary data available at https://doi.org/10.1088/1361-6560/ae1aca/data1.
